# Henoch-Schönlein purpura and drug and vaccine use in childhood: a case-control study

**DOI:** 10.1186/s13052-016-0267-2

**Published:** 2016-06-18

**Authors:** Liviana Da Dalt, Claudia Zerbinati, Maria Stefania Strafella, Salvatore Renna, Laura Riceputi, Pasquale Di Pietro, Paola Barabino, Stefania Scanferla, Umberto Raucci, Nadia Mores, Adele Compagnone, Roberto Da Cas, Francesca Menniti-Ippolito, the Italian Multicenter Study Group for Drug and Vaccine Safety in Children, Francesca Menniti-Ippolito, Roberto Da Cas, Giuseppe Traversa, Carmela Santuccio, Patrizia Felicetti, Loriana Tartaglia, Francesco Trotta, Pasquale Di Pietro, Paola Barabino, Salvatore Renna, Laura Riceputi, Pier-Angelo Tovo, Clara Gabiano, Antonio Urbino, Luca Baroero, Daniele Le Serre, Silvia Virano, Liviana Da Dalt, Chiara Stefani, Claudia Zerbinati, Giorgio Perilongo, Marco Daverio, Michela Maretti, Beatrice Galeazzo, Giulia Rubin, Stefania Scanferla, Elena Chiappini, Sara Sollai, Maurizio De Martino, Sabrina Becciani, Martina Giacalone, Simona Montano, Giulia Remaschi, Alessia Stival, Piera Abate, Ilaria Leonardi, Nicola Pirozzi, Umberto Raucci, Antonino Reale, Rossella Rossi, Nadia Mores, Giulia Bersani, Adele Compagnone, Antonio Chiaretti, Riccardo Riccardi, Costantino Romagnoli, Vincenzo Tipo, Michele Dinardo, Fabiana Auricchio, Teodoro Polimeno, Maria Colomba Bonagura, Alessandra Maccariello, Fortunata Fucà, Eleonora Di Rosa, Domenica Altavilla, Anna Mecchio, Teresa Arrigo

**Affiliations:** Department of Woman and Child Health, University of Padua, Padua, Italy; Pediatric Department, Treviso Hospital, Treviso, Italy; Giannina Gaslini Children Hospital, Genoa, Italy; Pediatric Emergency Department, Bambino Gesù Children’s Hospital, IRCCS, Rome, Italy; Pharmacology and Pediatrics, Università Cattolica S. Cuore, Rome, Italy; National Center for Epidemiology, Surveillance and Health Promotion, National Institute of Health, Viale Regina Elena, 299 - 00161 Rome, Italy

**Keywords:** Children, Adverse drug reaction, Henoch-Schönlein purpura, Vaccine

## Abstract

**Background:**

Henoch-Schönlein purpura (HSP) is the most common vasculitis in childhood; nevertheless, its etiology and pathogenesis remain unknown despite the fact that a variety of factors, mainly infectious agents, drugs and vaccines have been suggested as triggers for the disease. The aim of this study was to estimate the association of HSP with drug and vaccine administration in a pediatric population.

**Methods:**

An active surveillance on drug and vaccine safety in children is ongoing in 11 clinical centers in Italy. All children hospitalized through the local Paediatric Emergency Department for selected acute clinical conditions of interest were enrolled in the study. Data on drug and vaccine use in children before the onset of symptoms leading to hospitalization were collected by parents interview. A case-control design was applied for risk estimates: exposure in children with HSP, included as cases, was compared with similar exposure in children with gastroduodenal lesions, enrolled as controls. HSP cases were validated according to EULAR/PRINTO/PRES criteria. Validation was conducted retrieving data from individual patient clinical record.

**Results:**

During the study period (November 1999–April 2013), 288 cases and 617 controls were included. No increased risk of HSP was estimated for any drug. Among vaccines, measles-mumps-rubella (MMR) vaccine showed an increased risk of HSP (OR 3.4; 95 % CI 1.2–10.0).

**Conclusions:**

This study provides further evidence on the possible role of MMR vaccine in HSP occurrence.

## Background

Henoch-Schönlein purpura (HSP), recently renamed as IgA vasculitis, is a systemic leukocytoplastic vasculitis characterized by IgA1 dominant immune deposits [1, 2].

HSP is the most common vasculitis in childhood with an incidence of 10–20 cases per 100,000 in children under 17 years with a peak incidence of 70 cases per 100,000 in the 4–6 year age group [1, 2].

According to the last endorsed criteria by the EULAR/PINTO/PRES and American College of Rheumatology (ACR) the HSP diagnosis requires the presence of the palpable purpura and at least one of the following: arthritis or arthralgia, diffuse abdominal pain, renal involvement with haematuria and/or proteinuria or any biopsy showing predominant IgA deposition [2–4].

The HSP has an excellent outcome in the majority of cases with a complete remission in four weeks, about 20–55 % of children have a renal involvement but only <1 % develop an end-stage kidney disease. The pathogenesis of this vasculitis should be attributed to the IgA1 containing immune complexes deposition on the small vessels causing the damage and consequently all clinical manifestations. Although HSP clinical manifestations and prognosis are well-defined, the etiology of the disease remains unknown. It is clear that genetics and an abnormal immune response play a pivotal role in the pathogenesis of HSP [2, 5–7].

However a combination of additional factors has been suggested to trigger the disease, as infectious agents, drugs and vaccinations [8–14].

HSP following drug and vaccine administration has been described in case reports and in a small number of observational studies conducted during vaccination campaigns [15–34].

Since 1999 the Italian National Institute of Health is coordinating an active surveillance on the role of drugs and vaccines in the occurrence of specific clinical conditions responsible for hospitalization of pediatric patients. Non-infectious muco-cutaneous diseases are among the clinical conditions of interest and the present study focused on HSP cases to estimate their association with drug and vaccine use in the pediatric population.

## Methods

### Setting and study population

The Italian multicenter study on drug and vaccine safety in children involved 11 Italian Pediatric hospitals/wards spread throughout the country (Treviso, Padua, Naples, Genoa, Turin, Florence, Perugia, Palermo, Messina and Rome, with two centers).

Were enrolled in the study all children (age > 1 month and ≤ 18 years) hospitalized through the Emergency Departments (ED) for the following acute conditions: thrombocytopenia (platelet count <100 × 10^3^/L); acute non-infectious, non-febrile neurological disorders; endoscopically confirmed gastroduodenal lesions and/or clinically defined haematemesis and melena and non-infectious muco-cutaneous diseases and vasculitis. Exclusion criteria were represented by a concomitant diagnosis of cancer or immunodeficiency.

### Data collection

During hospital admission of the child, a trained pharmacist/physician interviewed parents to collect demographic and clinical information using a structured questionnaire.

Data on drug exposure in a time window of 3 weeks preceding hospitalization, extended to 12 weeks for vaccines, were collected.

For all children the inclusion in the study was based on the diagnosis retrieved from the ED records independently of drug and vaccine exposure.

### Ethical approval and consent to participate

According to the Italian regulation, retrospective observational studies are only required to be notified to ethical committees. The study protocol was notified to the ethical committee of each participating Center. Before parents interview, a written informed consent to use data for research purposes was obtained.

### Definition of cases and controls

All children hospitalized with a diagnosis of HSP at admission were included as cases. Discharge diagnosis was retrieved from clinical records and validated by clinicians, according to EULAR/PRINTO/PRES criteria for classification of HSP [3, 4]. Validation was conducted retrieving data from individual patient clinical record, blinded with respect to drug and vaccine exposure. Only validated cases were analyzed.

Children hospitalized for gastroduodenal lesions were considered as appropriate controls, since they represent an acute condition admitted through the EDs in the same clinical centers in which cases were identified.

### Statistical analysis

Descriptive analyses of demographic characteristics of case and control patients, drug and vaccine exposure were performed. Categorical variables, presented as numbers and percentages, were compared using the *Chi Square* test, while continuous variables, reported as median and range were compared using the Mann-Whitney *U* test. All tests were two sided and significance was set at *p* < 0.05.

A case-control study design was applied to compare drug and vaccine exposure in children with HSP, (cases) and children with gastroduodenal lesions (controls). A multiple logistic regression model was used to estimate adjusted Odds Ratios (ORs) and related 95 % confidence interval (CI). Age and concomitant use of any other drug were considered potential confounding factors. Statistical analysis was performed by means of IBM® SPSS® statistics (version 22).

## Results

**Fig. 1:**
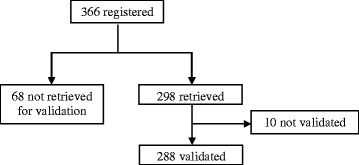
Enrollment, retrieving and validation process of HSP patients

During the period November 1999–April 2013, a total of 2600 patients with a diagnosis of muco-cutaneous disease were enrolled in the study. Among these hospitalized pediatric patients, 366 were diagnosed as HSP at the ED. For the validation phase of HSP cases, clinical records of 298 children (81 % of total), were retrieved. Only 10 cases did not fulfill the diagnostic criteria for the disease as described in the score according to EULAR/PRINTO/PRES criteria (Fig. 1). In all the remaining 288 HSP validated cases, a palpable purpura was present, accompanied by arthralgia/arthritis in 85 %; abdominal involvement with abdominal pain, melena, intussusception in 65 %; renal involvement with haematuria, proteinuria in 35 % and scrotal swelling in 7 %. In the same period 617 children were enrolled with gastroduodenal lesions. Haematemesis and melena accounted for most of gastrointestinal diagnosis. Characteristics of cases and controls are reported in Table 1.

**Table 1:** Characteristics of the study population

	Cases	Controls^a^	
Number	288	617	
Median age (range)	6y (2 m–18y)	3y (1 m–18y)	*p* < 0.001
% Females	48	45	*p* = 0.8
Median length of hospital stay (range, days)	4 (1–31)	3 (1–37)	*p* < 0.001
Median interval from drug/vaccine to symptom onset (range, days)	2 (0–60)	0 (0–90)	*p* < 0.001
Children exposed to drugs: *N* (%)	175 (61)	435 (71)	*p* < 0.08
Children exposed to vaccines: *N* (%)	21 (7)	68 (11)	*p* = 0.08

^a^gastroduodenal lesions

Median duration of hospitalization was similar in both groups, even if the statistical test was statistically significant, indicating difference in the distribution around the median. Controls were significantly younger than cases (6 vs. 3 years). Drug and vaccine exposure resulted significantly higher in controls.

Crude and adjusted ORs were estimated for each drug considering at least five exposed cases. No significant increased risk was estimated for any drug (Table 2).

**Table 2:** Odds ratio of developing HSP associated with drugs

Drugs	Cases (288)	Controls^a^ (617)	Crude OR (95 % CI)	Adjusted OR^b^ (95 % CI)
Antibiotics	65	156	0.7 (0.5–1.0)	1.0 (0.7–1.5)
Penicillins	38	72	0.9 (0.5–1.4)	1.3 (0.8–2.0)
Cephalosporin	21	49	0.7 (0.4–1.3)	1.1 (0.7–2.0)
Macrolides	7	37	0.3 (0.1–0.7)	0.4 (0.2–0.9)
NSAIDs	47	101	0.8 (0.5–1.2)	0.9 (0.6–1.4)
Niflumic acid	12	14	1.4 (0.6–3.3)	1.9 (0.6–1.4)
Ibuprofen	29	61	0.8 (0.5–1.3)	1.1 (0.7–1.7)
Paracetamol	62	163	0.6 (0.4–0.9)	0.8 (0.6–1.1)
Corticosteroids	31	104	0.5 (0.3–0.8)	0.8 (0.5–1.3)
Non users	113	182		

^a^gastroduodenal lesions ^b^OR adjusted by age and concomitant use of other drug

The risk estimated for HSP within 12 weeks after vaccination resulted higher, more than 3 times, for MMR vaccines with an OR of 3.4 (95 % CI 1.2–10.0) while no significant increased risks were observed for diphtheria, tetanus, acellular pertussis (DTaP) and any vaccine (Table 3).

**Table 3:** Odds ratio of developing HSP associated with vaccines

Vaccine	Cases (288)	Controls^a^ (617)	Crude OR (95 % CI)	Adjusted OR^b^ (95 % CI)
MMR	8 (2.8 %)	6 (1 %)	2.7 (0.8–9.7)	3.4 (1.2–10.0)
DTaP	4 (1.4)	6 (1 %)	1.4 (0.3–6.1)	2.0 (0.5–7.7)
Any vaccine	21 (7.3 %)	68 (11 %)	0.6 (0.4–1.1)	0.9 (0.5–1.4)

^a^gastroduodenal lesions ^b^OR adjusted by age

## Discussion

Despite the fact that HSP is the commonest, mainly self-limiting, systemic vasculitis in childhood, its etiology and pathogenesis remain still to be fully understood. Many chemical and infectious triggers have been recognized for the HSP typical vascular IgA deposition, including drugs and vaccines beside the role played by immunological, genetic and environmental factors [13, 14, 35]. Furthermore no risk estimates for HSP and drug or vaccine exposure have been published. This study aimed to investigate the association and the potential role of drugs and vaccines in the occurrence of HSP in a pediatric population.

HSP diagnosis is difficult at admission since there are no disease-specific laboratory abnormalities, neither signs and symptoms, histopathological findings are needed to properly identify HSP cases. Only HSP validated cases, according to the EULAR/PRINTO/PRES criteria, were considered for the statistical analyses. The causality assessment on each HSP case was not performed and was beyond the scope of this study.

The clinical presentation of the 288 validated cases confirmed what previously reported as main clinical features for HSP [2]. In addition to the palpable purpura, hallmark of the disease, articular and abdominal involvement has been described in the majority of the patients whereas renal and genital involvement were less frequent. According to the literature, the median age of HSP cases was in accordance with that reported elsewhere (4-6 years), so as the equal distribution among male and female subjects [1, 2, 5].

The characteristics of the 68 cases in which clinical records were not retrieved were similar for age, gender, previous febrile infections, drug and/or vaccine exposure to the retrieved cases thus excluding a possibility for selection bias.

Children hospitalized for gastroduodenal lesions have been considered as a suitable control group for HSP patients. In this study both cases and controls have been identified through EDs for an acute condition in the same clinical centers and, consequently, drug and vaccine exposure, preceding symptoms causing hospitalization, have been ascertained with the same procedure by interviewing parents during their child hospitalization.

Our results provide no evidence of an increased risk of developing HSP associated with any of the drugs considered.

A three-fold increase risk of developing HSP associated with MMR vaccine was estimated.

Prospective studies conducted during MMR vaccination campaign reported HSP cases following vaccine administration [28–30]. Within the Italian Pharmacovigilance System some spontaneous reports of HSP were reported after MMR vaccine [36]. During the Chinese MMR vaccination campaign, 30 severe adverse reactions out of 14.3 millions of administered doses were reported; among these, 28 cases were diagnosed as HSP with an estimated incidence of 2.1 per million/doses [30]. Our study confirmed these observational data, providing a risk estimate for this association.

Given the case-control design we could not calculate any incidence estimate.

This article confirms that HSP is a rare condition (288 children hospitalized in 14 years). Furthermore, the vaccinated cases were only 8, suggesting a very low absolute risk of the condition in children vaccinated with MMR vaccine. Thus, the benefit/risk profile of MMR vaccine is not affected by our results, being MMR vaccination an effective and safe tool against serious diseases in childhood.

With regard to drugs an association of gastroduodenal lesions with NSAIDs has been previously highlighted [37]. This could have lead to an underestimation of the results obtained in the present study concerning the role of NSAIDs in the development of HSP. It was beyond the objective of the present study to investigate the pathogenetic role of drugs and vaccines in the HSP occurrence.

## Conclusions

The association between MMR vaccination and HSP confirms previous published findings and adds a risk estimate. Further studies are needed to increase our understanding of the role of drugs and vaccines in the etiology of HSP, a disease with important effects on health of children for its potential, though rare, chronic outcomes.

## Abbreviations

HSP, Henoch-Schönlein purpura; Ig, Immunoglobulin
